# Dysfunctional self-reported interoception predicts residual symptom burden of fatigue in major depressive disorder: an observational study

**DOI:** 10.1186/s12888-023-05168-y

**Published:** 2023-09-13

**Authors:** Michael Eggart, Juan Valdés-Stauber, Bruno Müller-Oerlinghausen, Martin Heinze

**Affiliations:** 1 Faculty of Health Sciences, Brandenburg Medical School Theodor Fontane, Neuruppin, 16816 Germany; 2 Department of Psychiatry and Psychotherapy I, Ulm University and Center for Psychiatry Südwürttemberg, Ravensburg, 88214 Germany; 3 Faculty Social Work, Health and Nursing, Ravensburg-Weingarten University of Applied Sciences, Weingarten, 88250 Germany; 4 Faculty of Medicine and Psychology, Brandenburg Medical School Theodor Fontane, Neuruppin, 16816 Germany; 5 Charité – Universitätsmedizin Berlin, Berlin, 10117 Germany; 6 Department of Psychiatry and Psychotherapy, Brandenburg Medical School Theodor Fontane, Immanuel Klinik Rüdersdorf, Rüdersdorf, 15562 Germany

**Keywords:** Major depressive disorder, Interoception, Body awareness, Fatigue, Residual symptoms, Outcome predictors

## Abstract

**Background:**

Fatigue is a core symptom of major depressive disorder (MDD) and is frequently refractory to antidepressant treatment, leading to unfavorable clinical/psychosocial outcomes. Dysfunctional self-reported interoception (i.e., maladaptive focus on the body’s physiological condition) is prevalent in MDD and could contribute to residual symptom burden of fatigue. Therefore, we explored (a.) cross-sectional correlations between both dimensions and investigated (b.) prospective associations between interoceptive impairments at admission and symptom severity of fatigue at the end of hospitalization.

**Methods:**

This observational, exploratory study included 87 patients suffering from MDD who completed self-rating scales, the Multidimensional Assessment of Interoceptive Awareness, Version 2 (MAIA-2), and the Multidimensional Fatigue Inventory (MFI-20), at the beginning and end of hospitalization. Bivariate correlations (*r*) and hierarchical regression analyses were performed.

**Results:**

The cross-sectional analysis showed moderate to large negative correlations between the MAIA-2 and MFI-20 dimensions except for the *Not-Distracting* scale. Symptoms of general, physical, and mental fatigue at the end of hospitalization were predicted by reduced body *Trusting* (*β* = -.31, *p* = .01; *β* = -.28, *p* = .02; *β* = -.31, *p* = .00, respectively). Increased *Body Listening* (*β* = .37, *p* = .00), *Not-Worrying* (*β* = .26, *p* = .02), and diminished *Attention Regulation* (*β* = -.32, *p* = .01) predicted higher mental fatigue.

**Conclusions:**

Diminished body confidence at baseline identified patients at risk for post-treatment fatigue and could therefore serve as a target for improving antidepressant therapy. Body-centered, integrative approaches could address treatment-resistant fatigue in MDD. However, clinicians may also consider the potential adverse effect of increased *Body Listening* and *Not-Worrying* on mental fatigue in psychotherapeutic and counselling approaches. Due to the exploratory nature of this study, the results are preliminary and need to be replicated in pre-registered trials with larger sample sizes.

## Background

Fatigue is a core symptom of major depressive disorder (MDD) [1, 2] which includes “physical (e.g., reduced activity, low energy, tiredness, decreased physical endurance, increased effort with physical tasks and with overcoming inactivity, general weakness, heaviness, slowness or sluggishness, nonrestorative sleep, and sleepiness); cognitive (e.g., decreased concentration, decreased attention, decreased mental endurance, and slowed thinking); and emotional dimensions (e.g., decreased motivation or initiative, decreased interest, feeling overwhelmed, feeling bored, aversion to effort, and feeling low)” (p. 186) [3]. Cumulative evidence points to a high prevalence of fatigue in MDD, even in patients who respond to treatment, because energy-related symptoms are poorly addressed by antidepressants [4, 5]. Additionally, fatigue is a common adverse effect of antidepressants [6]. Symptoms of fatigue typically occur in the prodromal, acute, and residual phase of MDD but are considerably underrecognized despite their clinical and psychosocial relevance [7]. Prospective studies have shown the long-term detrimental effects of residual symptoms of fatigue following treatment which predict chronicity, faster relapse, or a recurrent course of depression [8, 9], diminished psychosocial and vocational functioning [10], suicidal ideation/attempts [11], and increased healthcare utilization [11]. Therefore, there is a clinical need to address the extent of fatigue during the initial treatment of MDD, to identify targets for new interventions, and to characterize patients under risk [5].

Interoception involves the sensation, interpretation, and integration of signals representing the physiological condition of the entire body [12–14] as well as subjective beliefs (i.e., top-down predictions) about homeostatic states [15]. Interoception is primarily involved in the afferent part of homeostatic/allostatic feedback loops that are inextricably linked to urges and motivational behavior [16], e.g. dehydration triggers thirst, which motivates water seeking and encourages water drinking. Beyond physiological regulation, a growing body of research has shown that bodily sensations contribute to affect, cognition, and social functioning [16–18]. The continuous integration of interoceptive information from the entire body has been essentially linked to self-awareness, which is underpinned by a “common sensation” (*German* Gemeingefühl) of bodily feelings that are existentially important to human subjectivity [12, 13, 19]. The sensation of physiological changes is a central component of influential theories of emotion [20–23], and impairments in interoceptive processing have been identified as correlates of numerous mental disorders and psychosomatic disturbances [14, 24]. A state of dysfunctional interoception is also a typical characteristic of MDD, emphasizing its importance for the study of mood disorders [25–31].

Several facets of interoception have been distinguished [32, 33], one of these is cardiac interoceptive accuracy which is commonly assessed by the heartbeat counting task (but note the critical discussion on its construct validity [34–36]). Using this task in depression research, cumulative evidence points to blunted heartbeat perception accuracy in moderately depressed persons compared to healthy controls [25–27]. In the present study, we refer to another facet of interoception, i.e. self-reported interoception, which comprises an individual’s disposition to be focused on interoceptive states (i.e., bodily feelings) by distinguishing between clinically maladaptive and beneficial attention styles toward the body including their regulative consequences [37, 38]. In the tradition of phenomenological psychopathology, depression (*latin* deprimere, to press down) is described as a primarily bodily experienced disorder characterized by somatic feelings such as pain, numbness, flu-like symptoms, heaviness, constrictions in parts of the body, oppressions diffusely extending over the whole body (e.g., globus feeling in the throat, armor vest or tire feeling around the chest, pressure in the head, leaden paralysis, diffuse anxiety), or rigidity aggravating to stupor [39, 40]. Hence, the lived body becomes a resistant material corpse in depression – an interoceptive process which has been termed “corporealization of the lived body” [41]. Previous research has demonstrated the clinical relevance of abnormal self-reported interoception as an independent predictor of residual symptoms of depression after hospital treatment-as-usual [28] and of overall treatment response [42].

Fatigue has been classified as a non-painful interoceptive feeling and ascribed to a state of dysfunctional interoception in MDD [14]. In a recent theory of fatigue, Stephan et al. conceptualized fatigue as an interoceptive response to a state of dyshomeostasis by referring to a hierarchical Bayesian framework [43]. Following this approach, dyshomeostasis involves a discrepancy between sensed (i.e., afferent inputs from viscera) and expected (i.e., homeostatic setpoints) physiological states over a prolonged period, leading to the occurrence of interoceptive prediction errors that signal failed efforts to regulate the internal milieu and thus could trigger the feeling of fatigue [43]. These mechanisms could explain the difference between fatigue and tiredness, the latter being seen as a deviation from homeostatic setpoints (e.g., elevated lactic acid concentrations after physical activity), which is successfully resolved by rest [43].

Previous studies investigating fatigue experience in MDD have primarily focused on monoamine neurotransmitters, neuronal circuits of energy-related systems, and on inflammatory processes, but without significant clinical consequences (for a review, see [3]). To the best of our knowledge, despite the theoretical proximity between interoception and fatigue [14, 43], associations between dysfunctional interoception and fatigue in depression have not been studied. Therefore, we sought to (1.) explore cross-sectional correlations between self-reported interoception and multidimensional fatigue, and (2.) to investigate prospective associations between interoceptive dysfunctions at admission and the occurrence of fatigue symptoms at hospital discharge in inpatients suffering from MDD.

## Methods

The study was approved by the ethics committee of Ulm University (registration number: 13/17). We followed the principles of the Declaration of Helsinki and obtained patient’s written informed consent.

### Procedure and participants

This observational, exploratory study involved a secondary analysis of data which were gathered in a longitudinal, naturalistic trial investigating the effects of self-reported interoception on treatment-as-usual outcomes in hospitalized patients suffering from MDD. Details on the study design, procedure of participant recruitment, inclusion/exclusion criteria, a study flow chart, and a synopsis of treatment components have been reported in the companion paper, which investigated interoceptive predictors of overall change in depression severity over the course of inpatient treatment [28]. A total of 87 patients who have been consecutively admitted to the depression ward of the Department of Psychiatry and Psychotherapy I (Ulm University, Center for Psychiatry Südwürttemberg, Weißenau, Germany) were included. The diagnosis of MDD was assessed by trained clinicians according to ICD-10 criteria [2]. Questionnaire data were collected within 48 h of hospital admission/discharge. Patients underwent guideline-based treatment-as-usual including psychotropic drugs, cognitive behavioral psychotherapy, depression-focused behavioral group therapy, mental health care nursing interventions (e.g., crisis intervention, progressive muscle relaxation according to Jacobson), dance movement therapy, physical activity and occupational therapy [28].

### Measures

#### Multidimensional Assessment of Interoceptive Awareness, Version 2 (MAIA-2)

The MAIA-2 [42, 44] is a self-administered questionnaire in the paper-and-pencil mode including 37 items to assess self-reported interoception on eight scales (brief scale descriptions, an item example, and internal consistency reliabilities for the present study are reported in brackets): 1.) *Noticing* (“awareness of uncomfortable, comfortable, and neutral body sensations”, e.g., “When I am tense I notice where the tension is located in my body”, McDonald’s *ω*_*pre*_ = .58, *ω*_*post*_ = .73); 2.) *Not-Distracting* (“tendency not to ignore or distract oneself from sensations of pain or discomfort”, e.g., “I try to ignore pain”, *ω*_*pre*_ = .67, *ω*_*post*_ = .70); 3.) *Not-Worrying* (“tendency not to worry or experience emotional distress with sensations of pain or discomfort”, e.g., “I can stay calm and not worry when I have feelings of discomfort or pain”, *ω*_*pre*_ = .67, *ω*_*post*_ = .70); 4.) *Attention Regulation* (“ability to sustain and control attention to body sensations”, e.g., “I can maintain awareness of my inner bodily sensations even when there is a lot going on around me”, *ω*_*pre*_ = .87, *ω*_*post*_ = .88); 5.) *Emotional Awareness* (“awareness of the connection between body sensations and emotional states”, e.g., “I notice that my body feels different after a peaceful experience”, *ω*_*pre*_ = .86, *ω*_*post*_ = .88); 6.) *Self-Regulation* (“ability to regulate distress by attention to body sensations”, e.g., “I can use my breath to reduce tension”, *ω*_*pre*_ = .76, *ω*_*post*_ = .84); 7.) *Body Listening* (“active listening to the body for insight”, e.g., “I listen to my body to inform me about what to do”, *ω*_*pre*_ = .77, *ω*_*post*_ = .84); 8.) *Trusting* (“experience of one’s body as safe and trustworthy”, e.g., “I am at home in my body”, *ω*_*pre*_ = .88, *ω*_*post*_ = .90). Analogous to Cronbach's *α*, the common rule of thumb cut-off value (*ω* ≥ .70) was considered adequate [45]. Participants were asked to circle one number on each item which applies to the current state (0 = never; 5 = always). Item scores (range: 0 = “never”; 5 = “always”) were averaged for each dimension taking into account reverse-scoring [42, 44].

Previous studies reported an eight-factor structure for MAIA and its revision MAIA-2, appropriate convergent/discriminant validity, and adequate internal consistency reliability except for two subscales (*Noticing* and *Not-Worrying*) [44, 46]. The instrument was also validated in a clinically depressed sample and showed good criterion validity to distinguish between treatment response groups [42]. A major strength of the MAIA-2 is its ability to differentiate between clinically maladaptive and beneficial attention styles towards interoceptive cues, except for the Noticing scale which is a unipolar measure of body awareness [37]. According to Mehling, maladaptive interoceptive attention, which is most evident in somatization or health anxiety, refers to a person’s dispositional tendency to focus on unpleasant body sensations in an anxious and hypervigilant manner associated with affective fragility [37]. For example, pain research has shown that pain-related anxiety and pain catastrophizing mediate the association between emotional instability and hypervigilance to pain, which in turn predicts increased pain intensity [47]. The initial conceptualization that low scores on the MAIA subscales correspond to maladaptive states of interoceptive attention [46] has recently been challenged by preliminary evidence showing that increased tendencies to Body Listening may also be clinically detrimental in some cases [48] (for further details, please see discussion below).

#### Multidimensional Fatigue Inventory (MFI-20)

The assessment of fatigue is limited to the reliance on validated self-report questionnaires either following an unidimensional or multidimensional approach that take into account physical, affective, and cognitive aspects [49]. The MFI-20 [49, 50] is a self-administered questionnaire in the paper-and-pencil mode including 20 items to assess subjective experience of multidimensional fatigue on five scales (brief scale descriptions, an item example, and internal consistency reliabilities for the present study are reported in brackets): 1.) *General Fatigue* (a person’s general expression about his/her functioning, e.g., “I feel fit”, *ω*_*pre*_ = .61, *ω*_*post*_ = .86); 2.) *Physical Fatigue* (somatic sensations which are related to tiredness, e.g., “Physically I feel I am in an excellent condition”, *ω*_*pre*_ = .79, *ω*_*post*_ = .88); 3.) *Mental Fatigue* (mental symptoms of fatigue such as lack of concentration and focus, e.g., “I can concentrate well”, *ω*_*pre*_ = .77, *ω*_*post*_ = .87); 4.) *Reduced Activity* (reduction in daily activity, e.g., “I think I do a lot in a day”, *ω*_*pre*_ = .81, *ω*_*post*_ = .87); 5.) *Reduced Motivation* (reduced motivation to start an activity, e.g., “I have a lot of plans”, *ω*_*pre*_ = .69, *ω*_*post*_ = .75). Participants were asked to check one box on each item which applies to the current state (1 = yes, that is true; 5 = no, that is not true). Item scores (range: 1 = “yes, that is true”; 5 = “no, that is not true”) were summed up for each dimension taking into account reverse-scoring [49]. High scale scores indicate dimensional symptom burden of fatigue. Previous research has demonstrated the factorial validity of the MFI-20, construct and convergent validity, and good internal consistency reliability in a clinical sample [49].

### Data analysis

The statistical analyses were performed in R 4.1.2 [51] using the packages *car* 3.0–12, *lm.beta* 1.5–1, *MBESS* 4.8.1, *psych* 2.1.9, *tidyverse* 1.3.1. The prevalence of post-treatment fatigue of any severity was estimated by referring to minimal clinically important difference scores, which yielded a two-point difference for each MFI-20 scale in a previous study (minimum sum-score = 4; cutoff: sum-score ≥ 6) [52]. Pearson’s product-moment correlations between self-reported interoception and multidimensional fatigue were investigated at two time points: a.) prior to (pre-treatment) and b.) at the end of hospital treatment (post-treatment). Multivariate associations were tested in a hierarchical regression analysis including two steps to determine the individual model contribution of self-reported interoception (block 1: inclusion of age, sex, body mass index, treatment duration, somatic comorbidity, antidepressant use, and pre-treatment fatigue; block 2: inclusion of MAIA-2 scales). Sociodemographic and clinical covariates were included according to general recommendations for observational studies [53] to account for potential confounding or suppression effects and for relevant predictors that have been shown to affect residual fatigue [5, 54]. The need to consider confounders when using the MAIA-2 has been discussed elsewhere [28, 55]. Multicollinearity was not detected (variance inflation factor for all predictors: < 5). The significance level was a priori set to *α* = .05. On the basis of previous research [28], G*Power 3.1.9.2 [56] was used to estimate the post hoc minimum sample size for hierarchical regression analysis which resulted in *N* = 85 (Cohen’s *f*^*2*^ = .20 [moderate effect], *α* = .05, 1-*β* = .80, number of tested predictors = 8; total number of predictors = 17).

## Results

Participant characteristics are shown in Table 1.

**Table 1:** Characteristics of included participants (*N* = 87)

Characteristics	*N* (%)	*M* ± *SD*
Age (years)	-	47.57 ± 10.64
Sex (female)	49 (56.32%)	-
Body mass index (kg/m^2^)		26.45 ± 5.29
School Education		
≤ 9 years	19 (21.84%)	-
10 years	40 (45.98%)	-
≥ 11 years	28 (32.18%)	-
Employment status		
Unemployed	21 (24.14%)	-
Employed	60 (68.97%)	-
Retired	6 (6.90%)	-
Main diagnosis (ICD-10)		
Single depressive episode (F32)	27 (31.03%)	-
Recurrent depressive disorder (F33)	60 (68.97%)	-
Severity of depression (ICD-10)		
Moderate (F3x.1)	8 (9.20%)	-
Severe without psychotic features (F3x.2)	79 (90.81%)	-
Number of previous psychiatric inpatient treatments	0: 33 (37.93%)	-
	1: 32 (36.78%)	-
	2: 11 (12.64%)	-
	> 3: 11 (12.64%)	-
Somatic comorbidity	27 (31.03%)	
Antidepressants		
SSRI	30 (34.48%)	-
SNRI	27 (31.03%)	-
TCA	17 (19.54%)	-
NASSA	18 (20.69%)	-
Treatment duration (weeks)	-	8.59 ± 4.24
MAIA-2 (baseline / posttreatment)		
Noticing	-	2.92 ± 0.96 / 3.32 ± 0.95
Not-Distracting	-	1.67 ± 0.76 / 2.08 ± 0.80
Not-Worrying	-	2.03 ± 0.92 / 2.50 ± 0.88
Attention Regulation	-	2.05 ± 0.99 / 2.74 ± 0.93
Emotional Awareness	-	3.35 ± 1.12 / 3.69 ± 0.84
Self-Regulation	-	1.66 ± 0.97 / 2.51 ± 0.99
Body Listening	-	1.56 ± 1.01 / 2.49 ± 1.04
Trusting	-	2.17 ± 1.22 / 3.05 ± 1.19
MFI-20 (baseline / posttreatment)		
General Fatigue	-	15.67 ± 3.27 / 10.94 ± 3.93
Physical Fatigue	-	14.77 ± 3.71 / 10.51 ± 4.17
Mental Fatigue	-	15.46 ± 3.45 / 10.89 ± 3.86
Reduced Motivation	-	13.94 ± 3.52 / 9.38 ± 3.43
Reduced Activity	-	15.25 ± 3.77 / 10.38 ± 3.83

*M* ± *SD* Mean ± standard deviation, *N* Absolute frequency, *%* Relative frequency, *BMI* Body mass index, *BDI-II* Beck Depression Inventory-II, *ICD-10* International Statistical Classification of Diseases and Related Health Problems (10^th^ revision), *MFI-20* Multidimensional Fatigue Inventory, *MAIA-2* Multidimensional Assessment of Interoceptive Awareness, Version 2, *NaSSA* Noradrenergic and specific serotonergic antidepressants, *SNRI* Serotonin–norepinephrine reuptake inhibitors, *SSRI* Selective serotonin reuptake inhibitors, *TCA* Tricyclic antidepressants

During the course of treatment, the severity of fatigue decreased significantly in all dimensions of the MFI-20: *General Fatigue*, mean change *ΔM* (T0-T1) = -4.72 (95% confidence interval [CI] -5.60, -3.85), *t*(86) = -10.76, *p* < .01, Cohen’s *d* = -1.15; *Physical Fatigue*, *ΔM* = -4.26 (95% CI -5.15, -3.83), *t*(86) = -9.62, *p* < .01, *d* = -1.03; *Mental Fatigue*, *ΔM* = -4.57 (95% CI -5.41, -3.74), *t*(86) = -10.93, *p* < .01, *d* = -1.17; *Reduced Motivation*, *ΔM* = -4.56 (95% CI -5.34, -3.75), *t*(86) = -11.14, *p* < .01, *d* = -1.19; *Reduced Activity*, *ΔM* = -4.87 (95% CI -5.85, -3.89), *t*(86) = -9.87, *p* < .01, *d* = -1.06. At the end of hospital treatment, the prevalence of patients reporting fatigue of any severity on each dimension was: *General Fatigue* 89.66% (*N* = 78); *Physical Fatigue* 89.66% (*N* = 78); *Mental Fatigue* 91.95% (*N* = 80); *Reduced Motivation* 88.51% (*N* = 77); *Reduced Activity* 86.21% (*N* = 75). Besides, significant improvements were detected for all the MAIA-2 subscales, which were reported in the companion paper [28].

Table 2 shows main findings for the bivariate associations between self-reported interoception and multidimensional fatigue. Moderate to large negative cross-sectional correlations were found between the dimensions of MAIA-2 and MFI-20 except for the *Not-Distracting* scale. The correlation coefficients were consistently higher for the post-treatment condition compared to pre-treatment states.

**Table 2:** Correlations between self-reported interoception and multidimensional fatigue (*N* = 87)

**MFI-20**	**General Fatigue**		**Physical Fatigue**		**Mental Fatigue**		**Reduced Motivation**		**Reduced Activity**	
**MAIA-2**	***r***_***pre***_	***r***_***post***_	***r***_***pre***_	***r***_***post***_	***r***_***pre***_	***r***_***post***_	***r***_***pre***_	***r***_***post***_	***r***_***pre***_	***r***_***post***_
**Noticing**	-.15	-.38^**^	-.04	-.29^**^	-.06	-.32^**^	-.09	-.39^**^	-.15	-.24^*^
**Not-Distracting**	-.04	-.14	-.11	-.17	.13	-.02	.03	-.12	.08	-.10
**Not-Worrying**	-.07	-.43^**^	-.21^*^	-.49^**^	-.24^*^	-.36^**^	-.21	-.40^**^	-.10	-.48^**^
**Attention Regulation**	-.32^**^	-.69^**^	-.30^**^	-.56^**^	-.44^**^	-.63^**^	-.33^**^	-.58^**^	-.34^**^	-.53^**^
**Emotional Awareness**	-.14	-.61^**^	-.10	-.51^**^	-.20	-.46^**^	-.08	-.59^**^	-.11	-.46^**^
**Self-Regulation**	-.19	-.70^**^	-.23^*^	-.70^**^	-.28^*^	-.49^**^	-.27^*^	-.58^**^	-.36^*^	-.59^**^
**Body Listening**	-.18	-.61^**^	-.22^*^	-.51^**^	-.18	-.51^**^	-.20	-.53^**^	-.26^*^	-.59^**^
**Trusting**	-.06	-.71^**^	-.23^*^	-.69^**^	-.20	-.61^**^	-.15	-.61^**^	-.24^*^	-.64^**^

*MAIA-2* Multidimensional Assessment of Interoceptive Awareness, Version 2, *MFI-20* Multidimensional Fatigue Inventory, *r*_*pre*_ Correlation coefficient (pretreatment), *r*_*post*_ Correlation coefficient (posttreatment)

^*^p < .05 (two-sided)

^**^p < .01 (two-sided)

In the multivariate analysis, symptoms of general, physical, and mental fatigue were prospectively predicted by baseline impairments in self-reported interoception (Table 3). Specifically, diminished *Trusting* in the body was associated with post-treatment symptoms of general, physical, and mental fatigue. Mental fatigue was also predicted by reduced capability to maintain attention to body sensations, the tendency towards a less worrying self-focus, and increased tendencies to listen to body sensations for insight. The MFI-20 dimensions *Reduced Motivation* and *Reduced Activity* were not predicted by baseline self-reported interoception. Longer duration of hospital treatment predicted reduced motivation at the end of treatment.

**Table 3:** Prediction of posttreatment residual fatigue by pretreatment self-reported interoception (*N* = 87)

Variable	General Fatigue					Physical Fatigue					Mental Fatigue					Reduced Motivation					Reduced Activity				
	***B***	***β***	***SE***	***t***	***p***	***B***	***β***	***SE***	***t***	***p***	***B***	***β***	***SE***	***t***	***p***	***B***	***β***	***SE***	***t***	***p***	***B***	***β***	***SE***	***t***	***p***
**Intercept**	5.54	.00	4.20	1.32	.19	3.24	.00	4.11	.79	.43	.12	.00	3.92	.03	.98	-.31	.00	3.56	-.09	.93	2.09	.00	4.47	.47	.64
**Age**	.01	.04	.04	.38	.71	.04	.10	.04	1.02	.31	.07	.21	.03	2.21	.03^*^	.08	.24	.03	2.28	.03^*^	.04	.10	.04	.88	.38
**Sex (ref.: female)**	.00	.00	.89	.00	.00	.17	.02	.90	.19	.85	-.28	-.04	.77	-.36	.72	1.23	.18	.78	1.59	.12	.03	.00	.97	.03	.98
**BMI**	.12	.17	.08	1.53	.13	.14	.18	.08	1.74	.09	.10	.14	.07	1.43	.16	.01	.02	.07	.16	.87	.14	.19	.09	1.55	.13
**Baseline Fatigue**	.35	.29	.13	2.66	.01^**^	.35	.32	.12	2.92	.00^**^	.37	.33	.12	3.17	.00^**^	.34	.35	.11	3.17	.00^**^	.23	.22	.13	1.73	.09
**Duration**	.09	.10	.10	.89	.38	.02	.02	.11	.21	.84	.15	.16	.09	1.69	.09	.22	.27	.09	2.38	.02^*^	.08	.09	.11	.72	.47
**Som. Comorbidity**	.88	.10	.92	.96	.34	1.56	.17	.95	1.64	.11	-.12	-.01	.78	-.15	.88	1.12	.15	.80	1.41	.16	.79	.10	.98	.80	.42
**SSRI**	-1.08	-.13	.99	-1.09	.28	-1.35	-.15	1.02	-1.32	.19	-.95	-.12	.86	-1.11	.27	.09	.01	.87	.11	.92	.27	.03	1.09	.25	.80
**SNRI**	.47	.06	1.02	.46	.64	.75	.08	1.04	.72	.47	.27	.03	.88	.31	.76	-.35	-.05	.89	-.39	.70	.71	.09	1.12	.63	.53
**TCA**	-.69	-.07	1.11	-.62	.53	.18	.02	1.12	.16	.87	.95	.10	.98	.97	.34	-.99	-.12	.97	-1.03	.31	.53	.06	1.19	.44	.66
**NASSA**	.17	.02	1.02	.17	.87	1.18	.11	1.04	1.13	.26	.33	.04	.88	.38	.71	-.51	-.06	.92	-.55	.58	.27	.03	1.11	.25	.81
**MAIA-2 N**	.32	.08	.68	.47	.64	-.03	-.01	.69	-.05	.96	-.35	-.09	.59	-.60	.55	.09	.02	.59	.15	.88	.17	.04	.73	.23	.82
**MAIA-2 ND**	-.15	-.03	.53	-.28	.78	-.49	-.09	.55	-.89	.38	-.58	-.12	.47	-1.25	.22	.35	.08	.47	.74	.46	-.53	-.11	.58	-.92	.36
**MAIA-2 NW**	-.35	-.08	.54	-.65	.52	-.16	-.03	.55	-.29	.78	1.09	.26	.47	2.30	.02^*^	-.19	-.05	.48	-.40	.69	.04	.01	.58	.06	.95
**MAIA-2 AR**	-.19	-.05	.57	-.32	.75	-.28	-.07	.57	-.50	.62	-1.26	-.32	.49	-2.56	.01^*^	.21	.06	.49	.43	.67	-.34	-.09	.61	-.56	.58
**MAIA-2 EA**	-.78	-.22	.58	-1.33	.19	-.51	-.14	.60	-.85	.40	.23	.07	.51	.44	.66	-.60	-.19	.51	-1.17	.25	-.25	-.07	.64	-.39	.69
**MAIA-2 SR**	-.59	-.15	.52	-1.15	.26	-.41	-.09	.53	-.76	.45	-.47	-.12	.45	-1.06	.29	.23	.06	.46	.50	.62	.00	.00	.57	.00	1.00
**MAIA-2 BL**	.89	.23	.55	1.61	.11	1.12	.27	.57	1.97	.05	1.42	.37	.48	2.97	.00^**^	.43	.13	.49	.87	.39	.99	.26	.60	1.65	.10
**MAIA-2 T**	-1.01	-.31	.39	-2.57	.01^*^	-.94	-.28	.40	-2.39	.02^*^	-.99	-.31	.33	-2.96	.00^**^	-.66	-.23	.34	-1.92	.06	-.77	-.24	.42	-1.83	.07
${{\varvec{R}}}_{{\varvec{a}}{\varvec{d}}{\varvec{j}}}^{2}$	22.66%					28.15%					40.03%					21.41%					4.09%				
***F(df)***	*F*(18, 68) = 2.40, *p* = .01^**^					*F*(18, 68) = 2.87, *p* = .00^***^					*F*(18, 68) = 4.19, *p* = .00^***^					*F*(18, 68) = 2.30, *p* = .01^**^					*F*(18, 68) = 1.20, *p* = .28				
**Δ** ${{\varvec{R}}}_{{\varvec{a}}{\varvec{d}}{\varvec{j}}}^{2}$** for MAIA-2**	11.86%					5.84%					15.50%					0.00%					-0.01%				
**Δ*F(df)* for MAIA-2**	Δ*F*(8, 68) = 2.46, *p* = .02^*^					Δ*F*(8, 68) = 1.77, *p* = .10					Δ*F*(8, 68) = 3.45, *p* = .00^***^					Δ*F*(8, 68) = .99, *p* = .45					Δ*F*(8, 68) = .87, *p* = .55				

*B* Unstandardized regression coefficient, *β* Standardized regression coefficient, *BMI* Body mass index (kg/m^2^), *df* Degrees of freedom, *F F*-test statistic, *MAIA-2* Multidimensional Assessment of Interoceptive Awareness, Version 2 (subscales: N = Noticing; ND = Not-Distracting; NW = Not-Worrying; AR = Attention Regulation; EA = Emotional Awareness; SR = Self-Regulation; BL = Body Listening; T = Trusting); ${R}_{adj}^{2}$ Adjusted coefficient of determination *R*^*2*^; *Δ* ${R}_{adj}^{2}$ *for MAIA-2* Change of adjusted *R*^*2*^ (individual contribution of MAIA-2 scales included as block 2), *SE* Standard error

^*^p < .05 (two-tailed); ^**^p < .01 (two-tailed); ^***^p < .001 (two-tailed)

## Discussion

In the present study, we found moderate to large correlations between self-reported interoception and burden of fatigue in depressed patients. Additionally, we have identified prospective predictors of post-treatment fatigue in a guideline-based therapy of MDD, which may help characterize patients at risk and improve current treatments. The main findings are discussed in the following sections.

First, the hypothesized associations between the experience of fatigue and facets of self-reported interoception [14, 43] have been confirmed by the cross-sectional analysis. We found moderate to large correlations which pointed in the expected directions, suggesting more dysfunctional bodily self-focus in patients reporting higher fatigue burden. The results also showed links between abnormal interoception and facets of fatigue that go beyond the physical dimensions, such as mental fatigue or motivation loss, which may be understood against the background of the ecological embodiment paradigm that embeds mental processes and psychopathology in sensorimotor experiences [57]. Our findings are consistent with a growing body of research implicating a reconceptualization of depression as a dysfunctional response to impaired interoception [26–28, 39, 41, 42, 58–60], which also challenges the prevailing assumptions about depression as a “brain disorder” [61]. Although the pathophysiology of fatigue has been associated with a state of dyshomeostasis that manifests as systemic low-grade inflammation [62], there is preliminary evidence against an inflammatory involvement in the etiology of dysfunctional self-reported interoception [55]. Research into the causal factors of disturbed interoception is still in its infancy [14].

Besides, we found a consistent pattern showing stronger post-treatment correlations than for pre-treatment. Following Goodwin & Leech [63], several factors affect the magnitude of Pearson’s *r* which will be briefly discussed: *a) “Is there a lack of variability in the data?” (p. 263–264).* This is evident for the MFI-20 scales *General Fatigue*, *Physical Fatigue*, and *Mental Fatigue*, which show restricted ranges (minimum score_pre_ > minimum score_post_) and smaller pre-treatment standard deviations. The increase in variance (contributing to covariance and the magnitude of correlation coefficient) is consequently explained by significant reductions in fatigue severity over the course of treatment in some patients, whereas others showed relatively high fatigue burden. In contrast, the MAIA-2 scales are more homogenous over both time points regarding their variability. These patterns are also observed in the scatterplots. *b) “Do the marginal distributions have dissimilar shapes?” (p. 264).* The skewness’ of the variables included in the correlational analysis are excellent (± 1.00) except for *Emotional Awareness*_*post*_ (skewness = -1.11; acceptable). Thus, difference between distributional shapes may not explain pre/post variability in *r*. *c) “Is there a nonlinear or curvilinear relationship between the two variables? (p. 264).* We checked scatterplots for all bivariate pairs and found no deviations from linearity. *d) “Are there one or more outliers in the dataset?” (p. 264).* When using boxplots together with scatterplots, we could not detect any influential outliers. *e) “Are there other unique characteristics of the sample that might be responsible for an unusually low value of r?” (p. 264).* The study included a relatively homogenous sample of severely depressed patients and did not recruit patients from ambulatory settings with lower fatigue symptom severity, which leads to selection bias. Pre-assessments could therefore be subject to insufficient effort responding on self-report measures (e.g., straight lining, random responding), which has been linked to psychiatric symptom burden, and inflates type II errors by decreasing the size of estimated correlations [64, 65]. *f) “Is the measurement reliability for either variable (or both) low?” (p. 264).* Carless responding may also affect internal consistency estimates [64], which could explain the lower pre-treatment scale reliabilities in our study. In summary, the unexpectedly low pre-treatment correlations are likely due to the interaction of lack of variability, pre-treatment sample characteristics, and measurement errors.

Second, diminished confidence in the body at baseline was identified as a prospective predictor of higher symptom severity of general, physical, and mental fatigue at the end of hospitalization. In particular, a lack of body trust has been shown as a common interoceptive abnormality in MDD [29, 66]. Additionally, a recent study has demonstrated the clinical importance of alleviating body mistrust in predicting overall response to treatment-as-usual in hospitalized MDD patients [42]. These findings are consistent with recently proposed theories which also ground the etiology of fatigue in interoceptive processes [43]. Stephan et al. [43] conceptualized fatigue and other symptoms of depression as a maladaptive response to chronic dyshomeostasis which cannot be resolved by allostatic regulation. Consequently, subjects develop beliefs about the uncontrollability and unpredictability of body sensations, which lead to “learned helplessness” [43] and probably to diminished body trust. The sense of low-self efficacy and diminished locus of control have a prominent history in depression research as contributing factors to MDD symptomatology [67]. These dysfunctional cognitions could also generalize to other situations [68] beyond the body, resulting in a global state of hopelessness [43]. However, previous research on the occurrence of learned helplessness has had a cognitive rather than somatic focus in terms of precipitating factors [67]. Moreover, the lack of body trust could reflect an existential dimension of the embodied human being (i.e., limited control over the body could lead to a threat to survival [43]), which can also be interpreted against the background of the phenomenological literature. Accordingly, the body is deprived of its predictable and sentient “material me” [12] and turns to an untrustworthy, inanimate corpse in depression [39, 41, 57], which can hardly enter into resonance with its environment [69], and loses sense of agency [70]. This fatigue-related “reification” of the body alters the person’s relation to the world, which is perceived *through* the felt body [40]: “it is the loss of an ordinarily taken for granted vitality that at the same time amounts to a draining away of practical possibilities from the experienced world” (p. 8).

Third, regarding residual symptoms of mental fatigue, we have shown that the ability to sustain attention to body sensations prospectively predicted less concentration difficulties after hospitalization. The close connection between targeted body focus and general ability to concentrate can be considered evident. We also found that increased listening to the body and an increased mindful cognitive appraisal of unpleasant body sensations at baseline is related to residual symptom burden in the mental fatigue dimension. These results contrast with the pattern of other interoceptive predictors studied, which show a negative relationship. It could be speculated to what extent too much focus on the body and an exaggerated mindful (i.e., non-judging) acknowledgment of unpleasant body sensations is associated with mental exhaustion. To the best of our knowledge, there is only one study reporting detrimental effects of heightened scores on the MAIA-2 *Body Listening* scale on clinical outcomes: Gioia et al. showed that increased tendency to listen to the body independently predicted the subsequent risk of suicidal ideation and nonsuicidal self-injury [48]. Preliminary findings thus suggest that increased active awareness of bodily sensations to gain insight, and possibly the adoption of an overly mindful cognitive style towards pain, may lead to unfavorable outcomes, calling into question the use of mindfulness-based intervention in depression therapy. These interventions are currently hyped [71], but have demonstrated unsatisfactory efficacy [72]. A growing body of research also points to rare but relatively severe and largely under-reported side effects [73, 74], which might also be better understood against the background of current evidence. Mindfulness training such as the “body scan” procedure focus on internal somatic signals, which at the same time also increases the tendency to listen to the body for insight and additionally modifies the cognitive appraisal of pain [75–77], which could be at the expense of mental energy. Clinicians should be aware of these effects to prevent an unfavorable outcome for the hospitalized patient.

Fourth, congruent with previous research, included participants reported a high prevalence of fatigue of any severity at the end of hospital treatment. Fava et al. summarized the prevalence of residual fatigue symptoms across clinical trials and found high proportions for all treatment response groups (partial responders: 63–98%; remission: 22–49%; partial response + remission: 85–91%) [5]. Among the many subtypes of MDD [78], fatigue is most frequently reported by patients suffering from atypical depression, which is characterized by a somatic symptom profile (leaden paralysis, hypersomnia, appetite changes etc.) [79]. Some authors speculated whether fatigue has a different underlying etiology than other symptoms of depression, which could explain its poor response to established treatments [3, 7]. Therefore, in the present study, a symptom-specific analysis of differentiated fatigue dimensions was performed instead of predicting the overall depression severity to account for the heterogeneity of MDD [80].

This study is subject to several limitations. Due to its observational nature, causal conclusions should not be drawn based on the present study findings. The findings are based exclusively on self-report measures of interoception and should be regarded as exploratory with potential risk for type I error as the analysis plan was not pre-registered. The ecological validity of our results may be limited by selection bias because we recruited in an academic hospital and therefore included a substantial proportion of severely depressed inpatients. Rather than using DSM-5®-based SCID-5-CV interviews, diagnoses were assessed by trained psychiatrists/psychologists following ICD-10 criteria [2]. In addition, the study was not adequately powered to examine predictors of residual fatigue exclusively in those who responded or partially responded to treatment—a project that should be considered in the future. Conceptual concerns were also raised, emphasizing the difficulty of distinguishing fatigue from anhedonia [1] and antidepressant-related side effects [5, 6]. The latter is not possible in the context of this observational study, as this would require a randomized placebo-controlled trial providing sufficient internal validity to distinguish between medication-induced and depression-related fatigue. In this respect, our results are uncertain as to whether self-reported interoception predicts medication side effects, depression-associated residual fatigue, or both. However, this tends to be of secondary importance for everyday clinical practice, since a large proportion of patients with major depression are treated with antidepressants. The MFI-20 *General Fatigue* and *Reduced Motivation* scales, and the MAIA-2 *Noticing*, *Not-Distracting* and *Not-Worrying* scales demonstrated inappropriate pre-treatment internal consistency reliabilities (*ω*_*pre*_ < .70), which may affect the validity of the findings regarding these scales. Future research should also include other facets of interoception, such as interoceptive accuracy, or composite measures such as the interoceptive state/trait prediction error. Furthermore, the individual effects of the treatment components in our naturalistic treatment-as-usual setting on self-reported interoception and multidimensional fatigue could not be identified in the current design. Randomized controlled trials are needed to address these challenges.

From a clinical standpoint, body sensations should be given greater consideration in clinical practice, both to identify patients at risk for adverse outcomes and to tailor personalized treatments. The prevention and treatment of residual symptoms of fatigue is not adequately addressed in available guidelines, although their clinical and psychosocial impact is increasingly recognized [4, 5]. Clinicians may consider discontinuing or reducing the dose of psychopharmaceuticals associated with residual fatigue, such as sedating tricyclic antidepressants, and should be aware of the high rate of treatment-related fatigue associated with selective serotonin reuptake inhibitors [5, 54, 81]. Exercise like graded aerobic training could be a promising treatment because it alleviates symptoms of residual fatigue [54]. An interoceptive mechanism of action has been assumed for exercise therapy [82]. However, the patient’s consent to participate in exercise therapy could be hampered by the main features of MDD, such as severe fatigue, lack of interest, motivation loss, psychosomatic complaints, or comorbidity with physical diseases [83]. Others have therefore suggested the use of psychoregulatory massage therapies as complementary treatments for MDD, which have shown antidepressant, anxiolytic, analgesic, calming, and fatigue-reducing effects, probably via an interoceptive mechanism of action [84–90]. The frequent use of touch-based treatments in depressed patients may support their wider application [91]. Besides, the effectiveness of massage therapy in reducing fatigue has been demonstrated in patients suffering from chronic fatigue syndrome [92], cancer [93], and multiple sclerosis [94].

## Conclusions

The burden of residual fatigue in depressed patients is not sufficiently addressed by current treatment guidelines, resulting in high prevalence rates even in treatment responders. Decreased confidence in the body at admission predicted increased fatigue severity at the end of hospital treatment and could therefore serve as a target for improving therapy. Despite increasing evidence, which recognizes the impact of fatigue on prognosis, daily functioning, and quality of life in MDD, targeted interventions are scarce and could be complemented by body-focused treatments. However, clinicians may also be aware of the potential negative effects of a) an increased tendency to listen to one’s body and b) the adoption of an overly mindful cognitive style towards pain on mental fatigue in terms of therapeutic plan, secondary prevention, and lifestyle counselling. Due to the exploratory nature of this study, our findings are preliminary and need to be replicated in pre-registered trials with larger sample sizes.

## Data Availability

The datasets used and/or analyzed during the current study are available from the corresponding author on reasonable request.
